# Catastrophic Sequalae Following Percutaneous Intervention in Case of Sigmoidectomy for Sigmoid Volvulus

**DOI:** 10.7759/cureus.15315

**Published:** 2021-05-29

**Authors:** Naveen Kumar Gaur, Oseen Shaikh, Suresh Chilaka, Chellappa Vijayakumar, Uday Kumbhar

**Affiliations:** 1 Surgery, Jawaharlal Institute of Postgraduate Medical Education and Research, Puducherry, IND

**Keywords:** inferior epigastric artery, percutaneous aspiration, hematoma, hemoglobin, ultrasonography

## Abstract

Injury to the inferior epigastric artery is infrequent and iatrogenic in most cases, which can be fatal and life-threatening in some cases due to unnoticed excessive hemorrhage. We present a 23-year-old male who underwent sigmoidectomy, end-to-end colorectal anastomosis with covering loop ileostomy for sigmoid volvulus. He developed intra-abdominal pus collection one week following surgery, for which ultrasound-guided aspiration was attempted. Post aspiration, the patient developed abdominal distension, pain with a significant drop in hemoglobin. Imaging showed active bleed from the branch of the inferior epigastric artery with massive intra-abdominal hematoma. The hematoma was evacuated, and the bleeding artery was identified and ligated. Postoperatively, there was no further drop in hemoglobin, and the patient was stable and hence discharged.

## Introduction

The inferior epigastric artery is a branch from the external iliac artery in most people; however, in few patients, the indirect origin from the external iliac artery has been documented [1,2]. Percutaneous needle aspiration is usually done to drain the intra-abdominal collection. Percutaneous aspirations are usually done under image guidance; however, there may be an injury to the vascular structure in some patients. Few patients may develop severe abdominal pain, tachycardia, sweating, and profound shock. Computed tomography angiography (CTA) is usually helpful for the diagnosis. Such patients can be treated by percutaneous vascular intervention like angioembolization or an open surgical approach and ligation of the bleeding vessel.

## Case presentation

A 23-year-old male patient presented to the emergency surgery team with complaints of diffuse abdominal pain, distension, bilious vomiting, and obstipation for two days. Radiological investigations were suggestive of large bowel obstruction due to Sigmoid Volvulus. The patient underwent sigmoidectomy and end-to-end colorectal anastomosis with covering loop ileostomy. On the third postoperative day, he was started on orals which he was tolerating well.

One week following surgery, he started developing multiple fever spikes. Ultrasound abdomen was done, which showed a localized collection of size 5.2 cm x 3.6 cm in the left iliac fossa for which ultrasound-guided percutaneous aspiration was attempted, and 10 mL of thick pus was drained. The patient started developing abdominal distension, severe abdominal pain, tachycardia, hypotension, and sweating few hours after the procedure.

**Figure 1:**
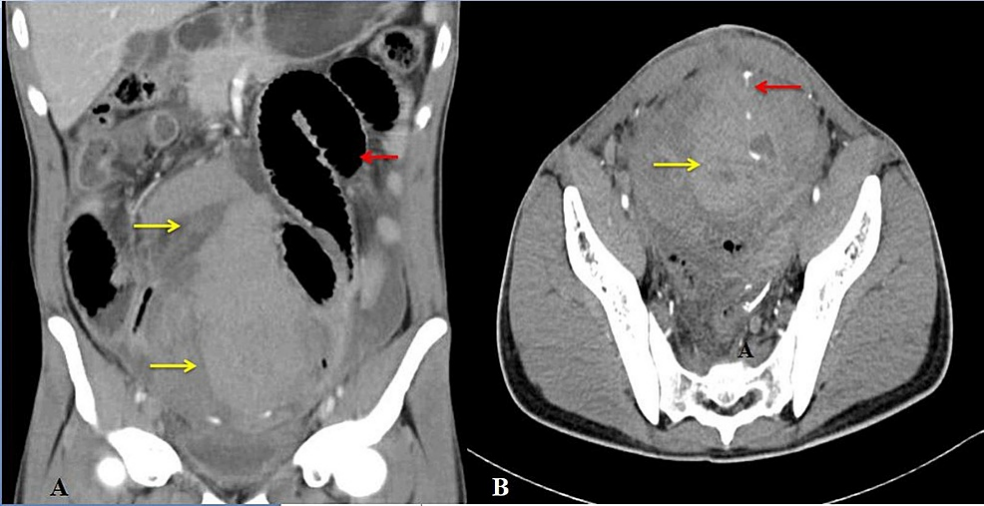
Contrast-enhanced computed tomography of the abdomen. (A) (Coronal view) Heterogeneously enhancing hematoma in abdomen and pelvis (yellow arrow) displacing bowel loops (red arrow). (B) (axial view) Heterogeneously enhancing hematoma in abdomen and pelvis (yellow arrow) with active contrast extravasation from the branch of the inferior epigastric artery from the abdominal wall (red arrow).

Blood investigations showed a significant drop in hemoglobin (from 10.6 g/dL to 7.1 g/dL). CTA showed 8.3 cm x 6.2 cm x 16.3 cm intraperitoneal hematoma extending from the pelvis, reaching up to the level of the lower pole of the right kidney. Hematoma appeared to be displacing the small bowel loops posteriorly. There was active contrast extravasation from the branch of the inferior epigastric artery suggestive of active bleed (Figures 1A, 1B).

**Figure 2:**
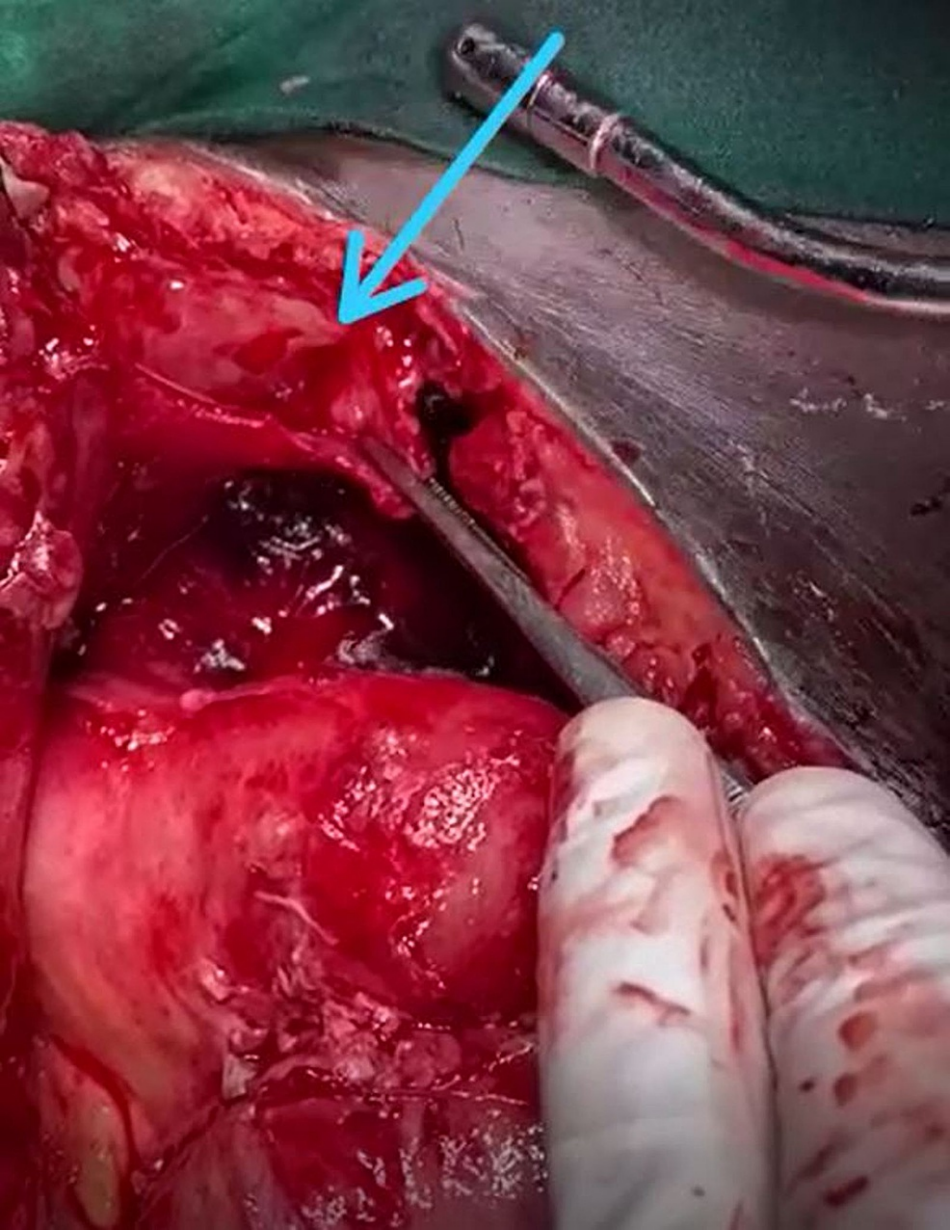
Intraoperative image showing active bleeding from the branch of the inferior epigastric artery from the abdominal wall (arrow).

Hence the patient underwent an emergency laparotomy. Intraoperatively, there was a massive intraperitoneal hematoma of approximately measuring 900 mL, which was evacuated. There was active spurting from a branch of the inferior epigastric artery from the anterior abdominal wall (Figure 2).

Bleeder was identified and ligated. The postoperative period was uneventful; hence the patient was discharged.

## Discussion

The mechanism of injury to the Inferior epigastric artery is iatrogenic in most cases, with paracentesis being the most common mechanism of injury [3]. Although paracentesis is considered to be a safe procedure, it is known to be associated with few complications [4]. Injury to the inferior epigastric artery or any branch arising from it can lead to significant bleeding, which can be life-threatening [5].

Another possible cause of inferior epigastric artery injury is the percutaneous fine-needle biopsy of abdominal organs, a beneficial, perhaps indispensable, diagnostic procedure done routinely in most hospitals. Added guidance utilizing radio-imaging modalities has ensured accurate placement of the biopsy needle and has led to widespread acceptance of the procedure. The rate of complications for percutaneous biopsy or aspiration depends not only on the size and type of needle but also on the number of attempts and blood coagulopathy [6-8]. There is an absolute difference in bleeding when one compares needles with various calibers in a patient without any coagulopathy [8].

Female patients are at double risk than males because of more retropubic vascular variations than males [9]. Patients of higher age are also at increased risk of vascular damage and bleed. The presence of the malignant disease has also been associated with higher risk [10]. Our patient underwent ultrasound-guided percutaneous aspiration of the intra-abdominal abscess following sigmoidectomy.

Patients develop pain abdomen and vomiting. Depending upon the amount of bleed, patients may develop tachycardia, hypotension, sweating, and a few may develop profound hemorrhagic shock. Clinically significant hemorrhage is defined as bleeding causing a drop of at least a 10-point hematocrit value and with hypotension or tachycardia, or there is a need for blood or blood products transfusion [3].

Imaging studies like CTA are helpful to localize the bleed as active contrast extravasations will be seen. There was active extravasation of the contrast from the branch of the inferior epigastric artery in our patient. Treatment of a patient who presents with inferior epigastric artery injury depends on the stability of the patient. If the patient is not stable, immediate laparotomy is mandated. Then, the active bleeder has to be identified and ligated. Another minimally invasive approach is angioembolization of the bleeding vessel. Our patient underwent immediate surgical exploration and ligation of the bleeding vessels to evacuate the intra-abdominal hematoma. This is the probable first case where the inferior epigastric artery got injured while doing percutaneous aspiration for intra-abdominal collections.

## Conclusions

Inadvertent puncture of any major vessel following percutaneous intervention for intra-abdominal collections in a postoperative patient can lead to life-threatening conditions. Therefore, early diagnosis has to be made based on the immediate clinical features of the patient. Early diagnosis and early intervention for the bleeding can reduce morbidity and fatality and prevent major interventions from being taken for such disasters. Emergent surgical intervention in such patients is life-saving and should be done at the earliest.

## References

[1] Kawai K, Honma S, Koizumi M, Kodama K (2008). Inferior epigastric artery arising from the obturator artery as a terminal branch of the internal iliac artery and consideration of its rare occurrence. Ann Anat.

[2] Sañudo JR, Mirapeix R, Rodriguez-Niedenführ M, Maranillo E, Parkin IG, Vázquez T (2011). Obturator artery revisited. Int Urogynecol J.

[3] Sobkin PR, Bloom AI, Wilson MW (2008). Massive abdominal wall hemorrhage from injury to the inferior epigastric artery: a retrospective review. J Vasc Interv Radiol.

[4] Runyon BA (1986). Paracentesis of ascitic fluid. A safe procedure. Arch Intern Med.

[5] Todd AW (2001). Inadvertent puncture of the inferior epigastric artery during needle biopsy with fatal outcome. Clin Radiol.

[6] Plecha DM, Goodwin DW, Rowland DY, Varnes ME, Haaga JR (1997). Liver biopsy: effects of biopsy needle caliber on bleeding and tissue recovery. Radiology.

[7] Welch TJ, Sheedy PF 2nd, Johnson CD, Johnson CM, Stephens DH (1989). CT-guided biopsy: prospective analysis of 1,000 procedures. Radiology.

[8] Bernardino ME (1984). Percutaneous biopsy. AJR Am J Roentgenol.

[9] Al-Talalwah W (2017). The inferior epigastric artery: anatomical study and clinical significance. Int J Morphol.

[10] McGill DB, Rakela J, Zinsmeister AR, Ott BJ (1990). A 21-year experience with major hemorrhage after percutaneous liver biopsy. Gastroenterology.

